# Effects of Extracorporeal Shock Wave Therapy in Patients with Mild-to-Moderate Carpal Tunnel Syndrome: An Updated Systematic Review with Meta-Analysis

**DOI:** 10.3390/jcm12237363

**Published:** 2023-11-28

**Authors:** Lei Zhang, Ting Yang, Long Pang, Yinghao Li, Tao Li, Chunsen Zhang, Lei Yao, Ran Li, Xin Tang

**Affiliations:** 1 Department of Rehabilitation, West China Hospital, Sichuan University, Chengdu 610041, China; 2 Operating Room of Anesthesia Surgery Center, West China Hospital, Sichuan University, Chengdu 610041, China; 3 West China School of Nursing, Sichuan University, Chengdu 610041, China; 4 Sports Medicine Center, West China Hospital, Sichuan University, Chengdu 610041, China; 5 Department of Orthopedics and Orthopedic Research Institute, West China Hospital, Sichuan University, Chengdu 610041, China

**Keywords:** extracorporeal shock wave therapy, carpal tunnel syndrome, local corticosteroid injection, systematic review, meta-analysis

## Abstract

Background: Carpal tunnel syndrome (CTS) is the most common entrapment syndrome, primarily affecting women between the ages of 40 and 70, and conservative treatments are the first option for mild-to-moderate CTS. However, the comparisons between extracorporeal shock wave therapy (ESWT) and other non-surgical methods in the treatment of mild-to-moderate CTS remain controversial, and an updated systematic review is needed. Methods: An electronic search was performed, and all available articles until August 2023 were included in the analysis. The overall quality of evidence was assessed by the GRADE approach. Meta-analyses were conducted using Manager V.5.3.3. Pooled effect sizes were expressed as the weighted mean difference (WMD) with 95% confidence intervals (CIs). Results: A total of 19 RCTs were included. Low-level quality evidence showed that ESWT outperformed the control intervention in terms of functional improvements, pain relief, electrodiagnostic parameters, and cross-sectional area of the median nerve at any time point of follow-up. Compared to local corticosteroid injection (LCI), there were statistically better improvements in functional improvements, pain relief, and electrodiagnostic parameters at 3 and 6 months of follow-up. Conclusions: There is low-level quality evidence to show that both fESWT and rESWT are more clinically effective than controls in symptom relief, functional enhancement, and electrophysiologic parameters’ improvement for patients with mild-to-moderate CTS at any time point of follow-up. Compared with LCI, ESWT yielded similar short-term (<1 month) but better medium- (1–6 months) and long-term (>6 months) improvements in pain relief and functional recovery with fewer potential complications.

## 1. Introduction

Carpal tunnel syndrome (CTS), the most prevalent entrapment syndrome, is estimated to affect 3% of individuals in the general population. It most frequently affects women between the ages of 40 and 70 [1,2]. CTS is typically idiopathic and results from persistently elevated pressure in the carpal tunnel and ischemia of the median nerve [3]. Some of the risk factors include diabetes mellitus, menopause, hypothyroidism, obesity, and repetitive wrist movements [4,5,6,7,8]. Early symptoms of CTS frequently include nocturnal pain, numbness, and tingling, which are normally followed by hypoesthesia in the wrists. Patients’ symptoms would worsen and interfere with their everyday activities in the absence of prompt diagnosis and treatment. Typical symptoms, physical examination, and electrodiagnostic testing are commonly used to diagnose CTS [2].

For mild-to-moderate CTS that has gone untreated in the past, conservative management is the first line of treatment. The most widely used treatments include nonsteroidal anti-inflammatory medications (NSAIDs), night splinting, local corticosteroid injection (LCI), and physiotherapies. If patients do not improve with conservative treatment, surgery should also be considered [2]. Even though LCI and surgery can both, in the majority of instances, result in favorable outcomes, there may be unintended repercussions or side effects, such as an increased risk of infection, poor tissue quality, weakness, and discomfort in the pillars [9].

A popular and non-invasive therapy is extracorporeal shockwave therapy (ESWT). It has a sequence of distinct sound impulses, a high-pressure peak, and a rapid pressure rise in a brief period of time. ESWT can be divided into focused ESWT (fESWT) and radial ESWT (rESWT) based on the variable therapeutic depth, area, and energy of the reflector [10]. ESWT is effective in treating a range of musculoskeletal illnesses due to its angiogenic, analgesic, and anti-inflammatory effects [11,12,13]. ESWT has been shown in numerous previous studies to improve functional outcomes and electrophysiologic parameters, hence reducing the symptoms of CTS [14,15]. However, some studies have revealed that it has a non-significant effect [16], raising questions about whether ESWT is a successful treatment for CTS. An updated systematic review with meta-analysis is necessary in light of the recent publication of numerous new randomized controlled trials (RCT).

The purpose of this study was to evaluate the effectiveness of EWST in the treatment of mild-to-moderate CTS and compare it with other conservative treatments. It is hypothesized that ESWT is equally as successful as other conservative methods such as LCI in the treatment of mild-to-moderate CTS.

## 2. Methods

This systematic review followed the preferred reporting items for systematic review and meta-analyses (PRISMA) statement [17].

### 2.1. Search Strategy

Two independent researchers (L.Z. and T.Y.) conducted separate searches of PubMed, EMBASE, the Cochrane Library, and Web of Science on 10 August 2023, using the following search terms: (Extracorporeal shock wave therapy OR Shock wave therapy OR Shock wave OR ESWT) AND (Carpal tunnel syndrome OR Median neuropathy OR Entrapment neuropathy OR Median neuritis). There was no time constraint for the publication date. All possible eligible studies investigating the effect of ESWT on CTS were manually retrieved. Any debated disagreement was resolved with a third researcher (X.T.).

### 2.2. Selection Criteria

Inclusion criteria: (1) patients with a confirmed diagnosis of mild-to-moderate CTS, and the severity of CTS was defined according to a modified scoring system [18], in which only the patients with a lack of sensory response, reduced, or even disabled movement, and abnormal distal motor latency are considered to have severe CTS.; (2) RCT comparing ESWT with other non-surgical interventions; (3) written in English. Exclusion criteria: (1) CTS caused by injury, tumor, infection, or systematic diseases; (2) presence of other diseases similar to carpal tunnel syndrome (such as cervical radiculopathy and polyneuropathy); (3) patients with previous injections or surgeries; (4) neither the pain nor the functional outcomes were reported.

### 2.3. Data Extraction

Two researchers (L.Z. and T.Y.) independently extracted data from the included studies. The characteristics of the enrolled studies (first author, year, country, sample size), and patients’ baseline information (age, sex, symptom duration, follow-up period, and intervention details) were meticulously recorded. The following clinical outcomes were extracted and pooled: (1) clinical symptoms and function assessed by the Boston Carpal Tunnel Questionnaire (BCTQ) [19]; (2) pain assessed by visual analog scale (VAS) [20]; and (3) electrodiagnostic evaluation, including the sensory nerve action potential (SNAP), compound muscle action potential (CMAP), sensory nerve conduction velocity (SNCV), sensory distal latency (SDL), motor distal latency (MDL), and cross-sectional area (CSA) of the median nerve [21]. Based on a systematic review of previous studies [22], we defined short-term as within 1 month after therapy, medium-term as 1 to 6 months after therapy, and long-term as 6 months or more after therapy.

### 2.4. Quality Assessment

Two investigators (L.Z. and T.Y.) independently assessed the methodological quality of each study using the revised Cochrane ROB-2 tool for RCTs [23]. Using the kappa statistics, inter-rater agreement was calculated (<0: less than chance agreement; 0.01–0.20: mild agreement; 0.21–0.40: fair agreement; 0.41–0.60: moderate agreement; 0.61–0.80: substantial agreement; 0.81–0.99: virtually perfect agreement) [24]. Discussions were continued in order to resolve any difference. Two authors (L.Z. and T.Y.) independently reviewed each study, and utilized the Cochrane Collaboration Grading of Recommendations Assessment, Development, and Evaluation (GRADE) approach to assess its evidence level. The inconsistency of results (downgraded by one level if significant heterogeneity was present by visual inspection or if the I^2^ value was greater than 50%), risk of bias (downgraded by one level if more than 25% of the participants were from studies with poor or unfair methodological quality), and imprecision of results (downgraded by one level if fewer than 70 participants were included in the comparison or downgraded by two levels if participants from pilot studies were included in the meta-analysis) were factors taken into consideration when determining the quality of the evidence for this study.

### 2.5. Data Synthesis and Analysis

Statistical evaluations were conducted using Manager V.5.3.3 (The Cochrane Collaboration, Software Update, Oxford, UK). We calculated the weighted mean difference (WMD) and pooled odds ratio (OR) with corresponding 95% confidence intervals (CIs) to assess the results. Statistical significance was defined as a *p* value < 0.05. Using Cochrane’s Q statistics and I^2^ statistics, we assessed and defined the heterogeneity of each qualified study. The data were synthesized using a random-effect model to account for the inescapable heterogeneity. Because the controlled interventions varied between studies, meta-analyses were conducted to compare: (1) ESWT and control; (2) ESWT and local corticosteroids injection (LCI). As for other interventions such as ultrasound therapy (US), only qualitative analyses were performed.

## 3. Results

### 3.1. Search Results and Study Characteristics

**Figure 1:**
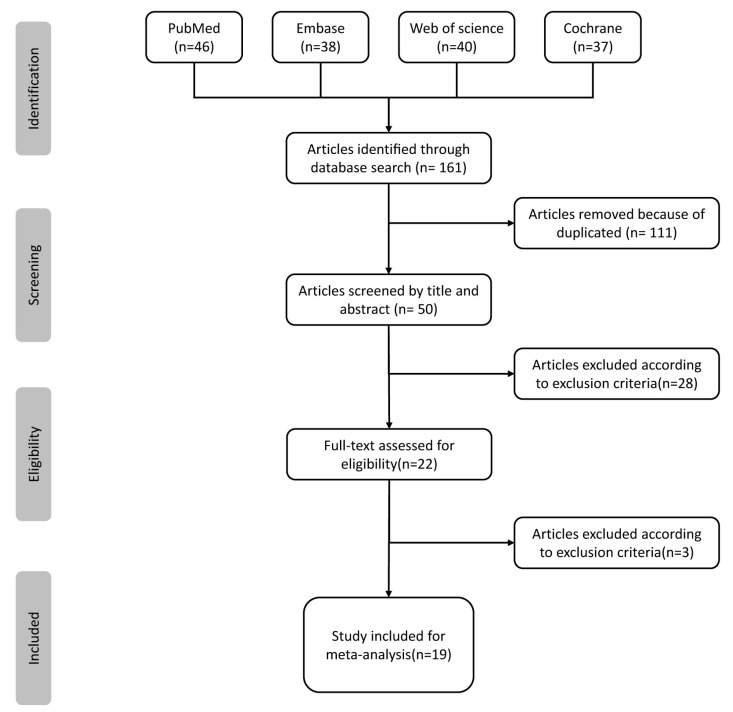
Flow chart of literature retrieval.

Two independent researchers (L.Z. and T.Y.) searched PubMed, Embase, Web of Science, and the Cochrane Library according to the identified keywords. All available articles until August 2023 were searched, and a total of 161 articles were retrieved. After 111 duplicates were removed, the titles and abstracts of the remaining articles were screened, and 28 articles were removed according to the inclusion and exclusion criteria. The remaining full texts of 22 articles were screened by two researchers (L.Z. and T.Y.) independently. Three studies assessing the effect of ESWT after carpal tunnel release were excluded. Finally, 19 RCTs [25,26,27,28,29,30,31,32,33,34,35,36,37,38,39,40,41,42,43] were included in this study (Figure 1). Table 1 shows the detailed characteristics of the included studies.

### 3.2. Risk of Bias in Included Studies

**Figure 2:**
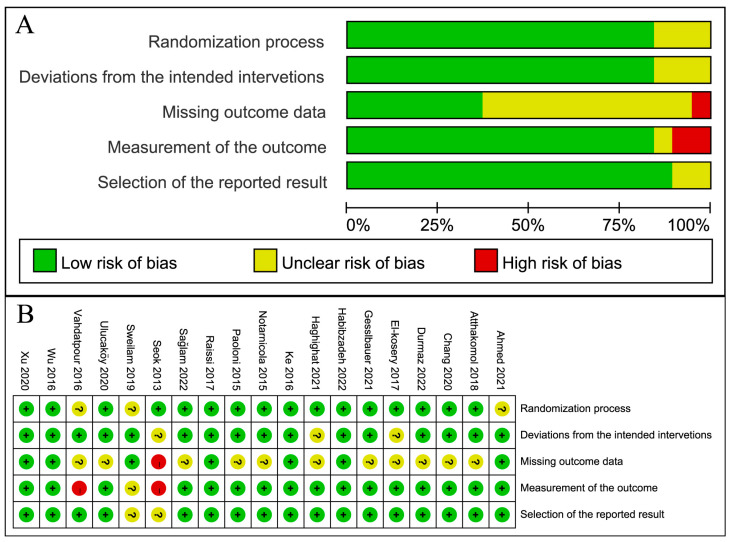
Risk of bias graph: (**A**) Graph of the risk of bias for the included studies; (**B**) graph of the risk of bias summary for the included studies [25,26,27,28,29,30,31,32,33,34,35,36,37,38,39,40,41,42,43]. “+”: low risk, “−”: high risk, “?”: unclear risk.

The risk of bias in the included studies was assessed by two independent reviewers (L.Z. and T.Y.). Inter-rater agreement was moderate for missing outcome data (=0.63), and moderate for measurement of the outcomes (=0.72), and good for the remaining domains (ranging from 0.81 to 0.90). The included studies were consistently low risk in the domains of the randomization process, deviations from the intended interventions, and selection of the reported outcomes. However, at least half of the studies were rated as an unknown risk of missing outcome data. Figure 2 summarizes the risk of bias in the eligible studies.

### 3.3. ESWT versus Control

**Figure 3:**
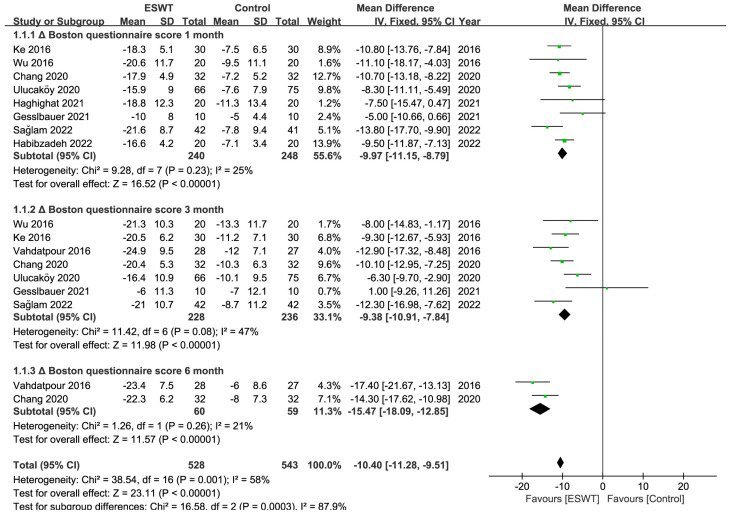
Meta-analysis of ESWT versus control: ΔBoston questionnaire score at 1-month, 3-month, and 6-month follow-up. The green squares represent the effect estimate of the individual studies and the horizontal lines indicate the confidence interval, and the dimension of the square reflects the weight of each study. The black diamond represents the combined point estimate and confidence intervals [27,29,30,31,32,33,38,41,42].

**Figure 4:**
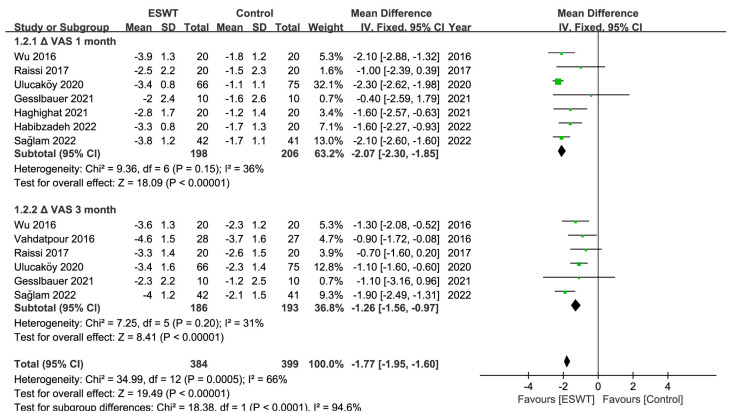
Meta-analysis of ESWT versus control: ΔVAS at 1-month and 3-month follow-up. The green squares represent the effect estimate of the individual studies and the horizontal lines indicate the confidence interval, and the dimension of the square reflects the weight of each study. The black diamond represents the combined point estimate and confidence intervals [29,30,31,33,37,38,41,42].

**Figure 5:**
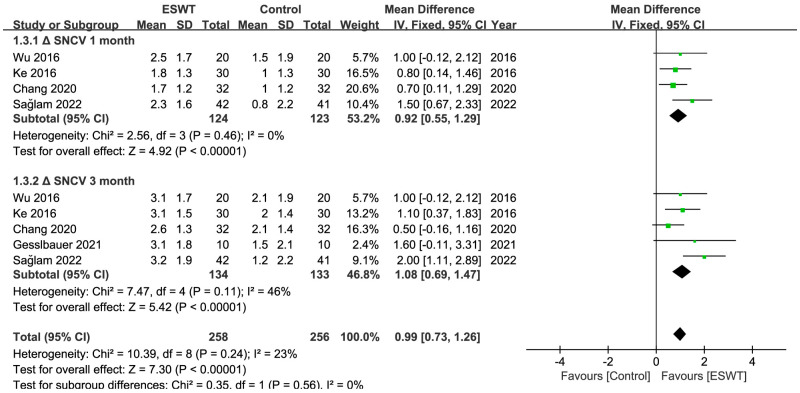
Meta-analysis of ESWT versus control: ΔSNCV at 1-month and 3-month follow-up. The green squares represent the effect estimate of the individual studies and the horizontal lines indicate the confidence interval, and the dimension of the square reflects the weight of each study. The black diamond represents the combined point estimate and confidence intervals [27,29,32,38,42].

**Figure 6:**
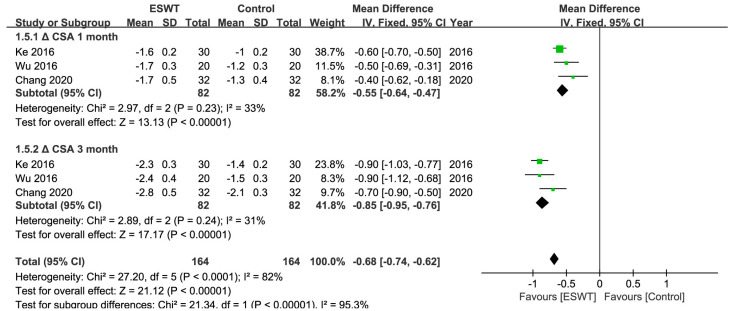
Meta-analysis of ESWT versus control: ΔCSA at 1-month and 3-month follow-up. The green squares represent the effect estimate of the individual studies and the horizontal lines indicate the confidence interval, and the dimension of the square reflects the weight of each study. The black diamond represents the combined point estimate and confidence intervals [27,32,42].

There were 10 RCTs [27,29,30,31,32,33,37,38,41,42] in total, involving 476 patients and at least 583 wrists. At any point throughout the follow-up period, the ESWT resulted in statistically greater improvements in the BCTQ score, VAS score, SNCV, MDL, and CSA compared to the control group (Table 2) (Figure 3, Figure 4, Figure 5 and Figure 6). Nevertheless, the quality of the evidence was reduced to low due to the possibility of bias, inconsistent outcomes, and imprecision (Table 3).

### 3.4. ESWT versus LCI

**Figure 7:**
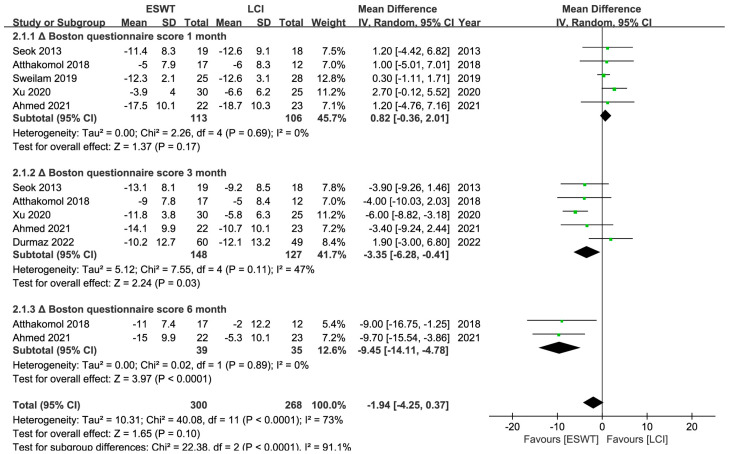
Meta-analysis of ESWT versus LCI: ΔBoston questionnaire score at 1-month, 3-month, and 6-month follow-up. The green squares represent the effect estimate of the individual studies and the horizontal lines indicate the confidence interval, and the dimension of the square reflects the weight of each study. The black diamond represents the combined point estimate and confidence intervals [25,26,39,40,43].

**Figure 8:**
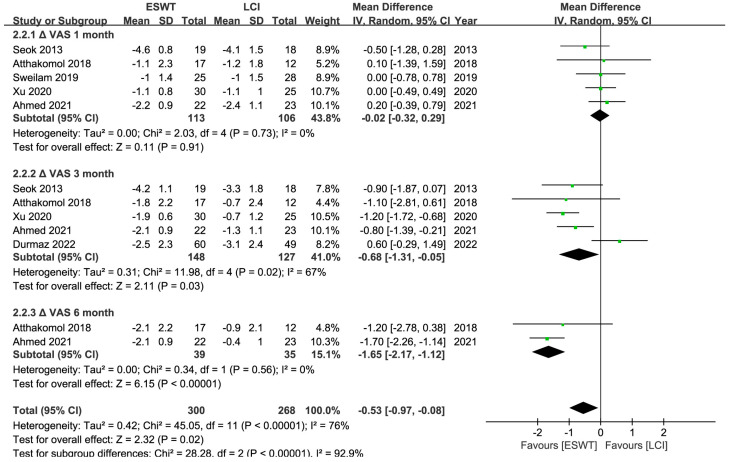
Meta-analysis of ESWT versus LCI: ΔVAS at 1-month, 3-month, and 6-month follow-up. The green squares represent the effect estimate of the individual studies and the horizontal lines indicate the confidence interval, and the dimension of the square reflects the weight of each study. The black diamond represents the combined point estimate and confidence intervals [25,26,35,39,40,43].

**Figure 9:**
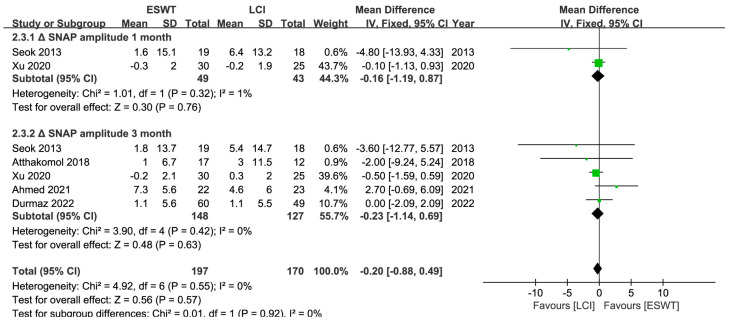
Meta-analysis of ESWT versus LCI: ΔSNAP at 1-month and 3-month follow-up; ΔCMAP at 1-month and 3-month follow-up. The green squares represent the effect estimate of the individual studies and the horizontal lines indicate the confidence interval, and the dimension of the square reflects the weight of each study. The black diamond represents the combined point estimate and confidence intervals [25,26,35,39,43].

**Figure 10:**
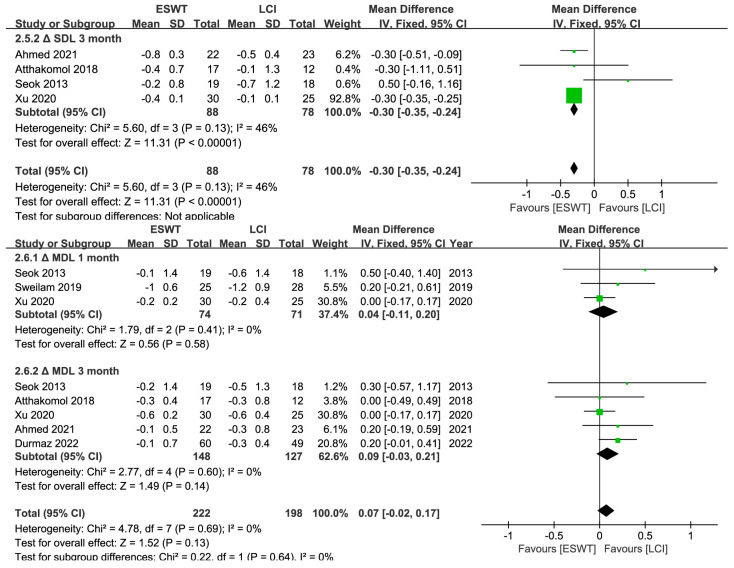
Meta-analysis of ESWT versus LCI: ΔSDL at 3-month follow-up; ΔMDL at 1-month and 3-month follow-up. The green squares represent the effect estimate of the individual studies and the horizontal lines indicate the confidence interval, and the dimension of the square reflects the weight of each study. The black diamond represents the combined point estimate and confidence intervals [25,26,35,39,40,43].

A total of 265 patients and at least 328 wrists were enrolled in 6 RCTs [25,26,35,39,40,43], and the ESWT led to comparable short-term (<1 month) but superior medium- (1–6 months) to long-term (>6 months) improvements in the BCTQ and VAS ratings. And ESWT yielded better medium-term improvements in the SDL than LCI, but no discernible difference could be seen in other electrodiagnostic parameters (Table 2) (Figure 7, Figure 8, Figure 9 and Figure 10). Similarly, the quality of evidence was reduced to low owing to the possibility of bias, inconsistency, and imprecision (Table 3).

### 3.5. ESWT versus Other Interventions

Only 2 RCTs [28,36] compared EWST with US for CTS, with 56 patients and at least 65 wrists included. Paoloni et al. [36] compared ESWT with cryo-US and US and discovered a notable improvement in pain and functionality across all groups. Moreover, patients in the ESWT group, as opposed to the US and cryo-US groups, experienced higher pain relief at the 12-week follow-up. El-Kosery et al. [28] also found that for postmenopausal women, ESWT was more effective in alleviating pain, increasing nerve conduction velocity, and lowering sensory and motor nerve delay. In another RCT [34], with 60 patients and at least 60 wrists included, ESWT was compared with a supplement diet mostly consisting of alpha lipoic acid (ALA), conjugated linoleic acid (GLA), and echinacea. This study came to the conclusion that ESWT relieved pain and enhanced functional abilities in CTS patients on par with nutritional supplements.

## 4. Discussion

In this study, we identified 19 RCTs with 856 patients and at least 1036 wrists to compare the clinical effectiveness of ESWT and other conservative methods for the treatment of mild-to-moderate CTS. The key finding of this study was that ESWT outperformed the control intervention in terms of BCTQ, VAS, SNCV, MDL, and CSA improvements at any time point of follow-up. Furthermore, after 3 and 6 months of follow-up, statistically better improvements in BCTQ, VAS, and SDL were observed when compared to LCI. However, no significant alterations in BCTQ and VAS were identified after the first month of treatment, and similar results in SNAP amplitude, CMAP amplitude, and MDL were found after 1 and 3 months of follow-up. Additionally, no adverse effects associated with ESWT treatment were identified in the included trials.

Previous systematic reviews [14,15,16] regarding the efficacy of ESWT on CTS yielded contradictory results. Kim et al. [14] found that ESWT could improve symptoms, functional outcomes, and electrophysiologic parameters in patients with CTS in 6 RCTs. Except for electrophysiologic measures, Xie et al. [15] aggregated data from 10 RCTs and found similar results. However, another systematic review by Chen et al. [16] concluded that ESWT provided limited therapeutic effects on CTS. Prior systematic reviews had several major flaws, including a limited number of included trials and patients, a lack of long-term follow-up (>6 months), a lack of subgroup analyses, and substantial heterogeneity in the interventions of the control group. With larger sample sizes, we concluded that both fESWT and rESWT could relieve pain and enhance function and electrophysiologic parameters in mild-to-moderate CTS patients from 1 to 6 months of follow-up.

Although the specific mechanism of ESWT in CTS is unknown, its anti-inflammatory and neural regeneration actions may account for symptom relief and functional improvement as compared to the control group. ESWT promotes the production of endothelial nitric oxide (NO) synthase in inflamed tissue, thereby increasing physiological levels of NO, which plays an important inhibitory function in the inflammatory response [11]. Reduced inflammation of the carpal canal could relieve symptoms by lowering the pressure around nerves. Ciampa et al. [44] found that extracorporeal shock waves rapidly boosted neuronal NO synthase activity and baseline NO outputs in the rat glioma cell C6 to achieve anti-inflammatory effects. Mense et al. [45] also found, using a rat model, that shockwave treatment with low-energy flux density could expedite the regeneration of injured nerve fibers, boosting the return of muscle sensitivity and functionality. Furthermore, animal studies have indicated that after ESWT treatment, neuronal regeneration could be activated by accelerating the clearance of damaged axons, boosting Schwann cell proliferation, and enhancing axonal regeneration. These mechanisms might interpret the improvements in function and electrodiagnostic parameters.

When compared with LCI, our pooled results demonstrated that the rESWT showed statistically worse function in the short-term follow-up, but better improvements in pain, function, and some electrophysiologic parameters after an adequate length of follow-up period. With only one included RCT comparing fEWST to LCI, no significant difference between these two interventions was found. In addition, Li et al. [46] conducted a meta-analysis with 5 RCTs included to compare the clinical effects between ESWT and LCI. Nonetheless, they reached the opposite conclusion that there were no significant differences in pain alleviation and functional enhancement between these two treatment options. As for electrophysiological parameters, ESWT was superior to LCI only regarding MDL, CMAP amplitude, and SNAP amplitude. Due to the chronic nature of CTS, they included only one trial with a fairly short follow-up period of 2 weeks, which was insufficient to compare the long-term efficacy, rate of recurrence, and potential consequences between these two medications. In contrast, we included more trials with longer follow-up periods to increase the sample size and extract more consistent outcome indicators to back up our findings.

Clinically, LCI, whose efficacy is supported by extensive clinical data [47], is becoming increasingly routinely utilized, particularly in patients with mild-to-moderate CTS. A systematic review [48], on the other hand, found that the incidence of severe adverse events (atrophy, hypopigmentation, or skin abnormalities) after soft-tissue LCI might reach 5.8%. According to Kaile et al. [9], the rate of potential adverse effects was reported to be 33% at around 6 weeks following 689 injections for CTS patients with 40 mg triamcinolone. The most prevalent symptom was short-term local pain, which occurred in 13% of injected limbs and disappeared within 3 weeks in all cases. Moreover, although the majority of side effects were temporary, 13 of the total hands had undergone permanent skin depigmentation or subcutaneous atrophy. Therefore, the frequency and course of this treatment should be strictly limited because of its potential complications. In contrast, as a non-invasive therapy technique, the ESWT group reported no major adverse effects in the studies included in this meta-analysis. In addition, the ESWT regimens in our study varied greatly, ranging from a single session to one session per week for four weeks. As a result, we could tailor the frequency and duration of ESWT to the patient’s needs while reducing the risk of consequences. Because ESWT could show both promising short-term efficacy and long-term accumulating therapeutic effects on CTS [49], the frequency and duration of ESWT can range from 1 to 4 sessions per week for a duration of 1 to 4 weeks, which could meet the needs of different groups of patients.

A limited number of RCTs [28,36] included in this study were carried out to compare the clinical effectiveness of ESWT and ultrasound therapy on mild and moderate CTS. They all demonstrated that ESWT and ultrasound therapy could be effectively used to reduce pain and improve function for patients with mild-to-moderate CTS in the short-term follow-up. Nonetheless, due to insufficient follow-up time, small sample size, and a lack of uniform outcome markers, more large-scale RCTs with long follow-up intervals are required to assess the long-term efficacy of these two therapies. Interestingly, Notarnicola et al. [34] performed an RCT to verify the efficacy of ESWT versus a nutraceutical consisting of alpha lipoic acid (ALA), conjugated linoleic acid (GLA), antioxidants, and Echinacea angustifolia for the treatment of CTS. They claimed that ESWT and a combination of ALA, GLA, and echinacea were both clinically efficacious for the treatment of CTS. This finding could be explained by the fact that ALA and GLA elicit antioxidant protective effects, thereby relieving pain and promoting axonal regeneration, while echinacea, which modulates the endogenous cannabinoid system, serves as an additional therapy effect. Similarly, more research with larger sample sizes of patients is needed.

This study has several limitations as follows. To begin, with 19 RCTs included, variability is unavoidable, which can be attributed to distinct types and therapeutic features of ESWT, variable follow-up periods, and diverse control interventions. Second, the BCTQ is made up of two main components: the symptom severity scale and the functional status scale. Nonetheless, due to insufficient data in the included studies, we were forced to analyze the overall BCTQ score, which may have resulted in some biases. Third, trials in our meta-analysis were followed up from 1 to 24 weeks. Longer observational periods, however, are needed to assess the long-term efficacy of ESWT in symptom alleviation and functional recovery. Finally, electrodiagnostic measures that only identify large myelinated nerves cannot assess the function of the small unmyelinated sensory nerves associated with CTS symptoms [46]. As a result, relevant research on the influence of ESWT on neurophysiology has a few limitations and should be further investigated.

## 5. Conclusions

There is low-level quality evidence to show that both fESWT and rESWT are more clinically effective than controls in symptom relief, functional enhancement, and electrophysiologic parameters improvement for patients with mild-to-moderate CTS at any time point of follow-up. Compared with LCI, ESWT yielded similar short-term (<1 month) but better medium- (1–6 months) and long-term (>6 months) improvements in pain relief and functional recovery with fewer potential complications. However, given the study’s limitations, further large RCTs with more stringent designs and longer follow-up periods are required for clear results.

## Figures and Tables

**Table 1:** Characteristics of the included studies.

Author	Year	Country	Wrists (Patients), n		Age, Years Mean ± SD		Sex (M/F)		Symptom Duration, Weeks Mean ± SD		Follow-up, Weeks	ESWT Protocol					Non-ESWT Protocol
			ESWT	Non-ESWT	ESWT	Non-ESWT	ESWT	Non-ESWT	ESWT	Non-ESWT		Intervention	Frequency (Hz)	Intensity	Total Shocks	Sessions (Time Period)	
**ESWT versus Control**																	
Ke, M.J., et al. [32]	2016	China	30 (23)	30 (23)	56.33 ± 1.48	58.13 ± 1.13	24/6	25/5	34.27 ± 5.85	35.34 ± 7.45	4, 10, 14	rESWT + night splint	5	4 bar	2000	3 (3 weeks)	Sham rESWT + night splint
Vahdatpour, B., et al. [41]	2016	Iran	NA (28)	NA (27)	51.5 ± 8.5	49 ± 7.3	NA	NA	14 ± 1.39	17.86 ± 2.14	12, 24	fESWT + night splint + drugs	3	0.05, 0.07, 0.1, 0.15 mJ/mm^2^	800, 900, 1000, 1100	4 (4 weeks)	Sham fESWT + night splint + drugs
Wu, Y.T., et al. [42]	2016	China	20 (17)	20 (17)	54.7 ± 7.96	57.8 ± 6.51	18/2	17/3	34.1 ± 33.11	36.1 ± 30.8	1, 4, 8, 12	rESWT + night splint	5	4 bar	2000	3 (3 weeks)	Sham rESWT + night splint
Raissi, G.R., et al. [37]	2017	Iran	NA (20)	NA (20)	46.1 ± 1.95	46.65 ± 2.23	18/2	19/1	NA	NA	3, 8, 12	rESWT + night splint	6	1.5 bar	1000	3 (3 weeks)	Night splint
Chang, C.Y., et al. [27]	2020	China	32 (20)	32 (20)	56.47 ± 1.41	58.63 ± 1.72	29/3	30/2	63.5 ± 7.55	61.34 ± 15.48	4, 12, 24	rESWT + PRP	5	4 bar	2000	1	Sham rESWT + PRP
Koçak Ulucaköy, R., et al. [33]	2020	Turkey	66 (47)	75 (50)	48.4 ± 10.1	48.5 ± 9.8	39/8	47/3	33.7 ± 38.1	24.8 ± 31.5	4, 12	rESWT + night splint	5	0.05 mJ/mm^2^	1000	3 (3 weeks)	Sham rESWT + night splint
Gesslbauer, C., et al. [29]	2021	Austria	NA (10)	NA (10)	55.8 ± 4.66	54 ± 17.4	8/2	6/4	29 ± 32.89	33.6 ± 44.26	3, 12	fESWT + night splint	4	0.05 mJ/mm^2^	500	3 (3 weeks)	Sham fESWT + night splint
Haghighat, S., et al. [31]	2021	Iran	NA (20)	NA (20)	50.1 ± 8.14	51.13 ± 9.06	18/2	14/6	NA	NA	4, 8	fESWT + night splint + drugs	3	0.05, 0.07, 0.1, 0.15 mJ/mm^2^	800, 900, 1000, 1100	4 (4 weeks)	Night splint + drugs
Habibzadeh, A., et al. [30]	2022	Iran	NA (20)	NA (20)	45.54 ± 11.90	51 ± 7.77	18/2	15/5	NA	NA	1, 4	rESWT + physiotherapy + night splint + drugs	6	1.5 bar	1500	4 (10 weeks)	Physiotherapy + night splint + drugs
Sağlam, G., et al. [38]	2022	Turkey	42 (32)	41 (32)	53.8 ± 11.8	53.4 ± 10.9	34/8	29/12	11.4 ± 11.1	10.6 ± 6.7	3, 12	rESWT + night splint + exercise	5	4 bar	2000	3 (3 weeks)	Night splint + exercise
**ESWT versus LCI**																	
Seok, H., et al. [39]	2013	Korea	19 (15)	18 (16)	54.03 ± 19.47	49.67 ± 18.83	12/3	14/2	9.76 ± 3.57	10.15 ± 2.3	4, 12	fESWT	6	0.09–0.29 mJ/mm^2^	1000	1	1 mL lidocaine + 1 mL triamcinolone acetonide (40 mg)
Atthakomol, P., et al. [26]	2018	Thailand	17 (13)	12 (12)	46 ± 9	53 ± 12	8/5	11/1	25.1 ± 19.3	26.4 ± 17.9	1, 4, 12, 24	rESWT	15	4 bar	5000	1	1 mL lidocaine + 1 mL triamcinolone (10 mg)
Sweilam, G., et al. [40]	2019	Egypt	NA (25)	NA (28)	37.6 ± 8.5	36.8 ± 8.8	21/4	23/5	25.1 ± 19.3	26.4 ± 17.9	2, 4	fESWT	10	2 bar	2500	2 (2 weeks)	1 mL triamcinolone acetonide (40 mg)
Xu, D., et al. [43]	2020	China	NA (30)	NA (25)	47.2 ± 1.86	46.9 ± 1.76	25/5	21/4	2.9 ± 0.8	2.8 ± 0.7	3, 9, 12	rESWT	6	1.5 bar	1000	3 (3 weeks)	1 mL lidocaine + 1 mL betamethasone (40 mg)
Ahmed, L., et al. [25]	2021	Egypt	22 (20)	23 (20)	51 ± 6	49 ± 8	16/4	15/5	NA	NA	4, 12, 24	rESWT	15	4 bar	5000	1	1 mL lidocaine + 1 mL triamcinolone (10 mg)
Öztürk Durmaz, H., et al. [35]	2022	Turkey	60 (33)	49 (28)	51.1 ± 7.1	54.1 ± 9.6	23/10	20/8	19.2 ± 24.1	17.4 ± 20.3	1, 12	rESWT	5	4 bar	2000	3 (3 weeks)	1 mL methylprednisolone (40 mg)
**ESWT versus US**																	
Paoloni, M., et al. [36]	2015	Italy	12 (8)	13 (8)	59.1 ± 12.5	56.5 ± 9.4	11/1	12/1	5.3 ± 3.1	5.1 ± 4.3	4, 12	fESWT	NA	0.05 mJ/mm^2^	2500	4 (3 weeks)	15 sessions of US
El-Kosery, S.M., et al. [28]	2017	Egypt	NA (20)	NA (20)	54.55 ± 2.3	54.5 ± 2.23	20/0	20/0	NA	NA	4	fESWT + night splint	NA	4 bar	2000	3 (4 weeks)	12 sessions of US + night splint
**ESWT versus nutraceutical**																	
Notarnicola, A., et al. [34]	2015	Italy	NA (34)	NA (26)	57.1 ± 9.5	60.2 ± 6.6	NA	NA	12.05 ± 1.43	11.92 ± 1.83	4, 8, 16, 24	fESWT	4	0.03 mJ/mm^2^	1600	3 (3 weeks)	A diet supplementary composed mainly of ALA, GLA, and echinacea

M, male; F, female; NA, not available; ESWT, extracorporeal shockwave therapy; LCI, local corticosteroid injection; US, ultrasound; fESWT, focused extracorporeal shockwave therapy; rESWT, radial extracorporeal shockwave therapy; PRP, platelet-rich plasma; ALA, alpha lipoic acid; GLA, conjugated linoleic acid.

**Table 2:** Summary of outcomes and subgroup analyses.

	No. of Studies (No. of Wrists)	WMD	95% CI	*I* ^2^	*p* Value	Comparison
**ESWT versus Control**						
Short-term follow-up (<1 month)						
**BCTQ**						
Overall	8 (488)	−9.97	−11.15, −8.79	25%	<0.01	ESWT > control
rESWT	6 (428)	−10.26	−11.48, −9.04	13%	<0.01	rESWT > control
fESWT	2 (60)	−5.84	−10.45, −1.22	0%	0.01	fESWT > control
**VAS**						
Overall	7 (404)	−2.07	−2.30, −1.85	36%	<0.01	ESWT > control
rESWT	5 (344)	−2.12	−2.35, −1.89	34%	<0.01	rESWT > control
fESWT	2 (60)	−1.41	−2.29, −0.52	0%	<0.01	fESWT > control
**SNCV**						
Overall	4 (247)	0.92	0.55, 1.29	0%	<0.01	rESWT > control
rESWT	4 (247)	0.92	0.55, 1.29	0%	<0.01	
**CSA**						
Overall	3 (164)	−0.55	−0.64, −0.47	33%	<0.01	rESWT > control
rESWT	3 (164)	−0.55	−0.64, −0.47	33%	<0.01	
Medium-term follow-up (1–6 month)						
**BCTQ**						
Overall	7 (464)	−9.38	−10.91, −7.84	45%	<0.01	ESWT > control
rESWT	5 (389)	−9.16	−10.81, −7.50	20%	<0.01	rESWT > control
fESWT	2 (75)	−6.75	−20.28, 6.78	83%	0.33	fESWT = control
**VAS**						
Overall	6 (379)	−1.26	−1.56, −0.97	31%	<0.01	ESWT > control
rESWT	4 (304)	−1.30	−1.78, −0.81	53%	<0.01	rESWT > control
fESWT	2 (75)	−0.93	−1.69, −0.16	0%	0.02	fESWT > control
**SNCV**						
Overall	5 (267)	1.08	0.69, 1.47	46%	<0.01	ESWT > control
rESWT	4 (247)	1.12	0.48, 1.75	58%	<0.01	rESWT > control
fESWT	1 (20)	1.60	−0.11, 3.31	NA	0.07	fESWT = control
**MDL**						
Overall	4 (179)	−0.29	−0.34, −0.24	20%	<0.01	ESWT > control
rESWT	2 (104)	−0.23	−0.42, −0.04	67%	0.02	rESWT > control
fESWT	2 (75)	−0.26	−0.47, −0.04	0%	0.02	fESWT > control
CSA						
Overall	3 (164)	−0.85	−0.95, −0.76	31%	<0.01	rESWT > control
rESWT	3 (164)	−0.85	−0.95, −0.76	31%	<0.01	
Long-term follow-up (>6 month)						
BCTQ						
Overall	2 (119)	−15.47	−18.09, −12.85	21%	<0.01	ESWT > control
rESWT	1 (64)	−14.30	−17.62, −10.98	NA	<0.01	rESWT > control
fESWT	1 (55)	−17.40	−21.67, −13.13	NA	<0.01	fESWT > control
**ESWT versus LCI**						
Short-term follow-up (<1 month)						
**BCTQ**						
Overall	5 (219)	0.82	−0.36, 2.01	0%	0.17	ESWT = LCI
rESWT	3 (129)	2.21	−0.14, 4.56	0%	0.07	rESWT = LCI
fESWT	2 (90)	0.35	−1.02, 1.72	0%	0.61	fESWT = LCI
**VAS**						
Overall	5 (219)	−0.02	−0.32, 0.29	0%	0.91	ESWT = LCI
rESWT	3 (129)	0.08	−0.28, 0.45	0%	0.66	rESWT = LCI
fESWT	2 (90)	0.35	−1.02, 1.72	0%	0.37	fESWT = LCI
**SNAP**						
Overall	2 (92)	−0.13	−0.90, 0.65	0%	0.75	ESWT = LCI
rESWT	1 (55)	−0.10	−1.13, 0.93	NA	0.85	rESWT = LCI
fESWT	1 (37)	−4.80	−13.93, 4.33	NA	0.30	fESWT = LCI
**CMAP**						
Overall	3 (145)	0.01	−0.16, 0.18	0%	0.92	ESWT = LCI
rESWT	1 (55)	0.00	−0.17, 0.17	NA	1.00	rESWT = LCI
fESWT	2 (90)	0.47	−0.82, 1.75	0%	0.48	fESWT = LCI
**MDL**						
Overall	3 (145)	0.04	−0.11, 0.20	0%	0.41	ESWT = LCI
rESWT	1 (55)	0.00	−0.17, 0.17	NA	1.00	rESWT = LCI
fESWT	2 (90)	0.25	−0.12, 0.62	0%	0.19	fESWT = LCI
Medium-term follow-up (1–6 month)						
**BCTQ**						
Overall	5 (275)	−3.94	−5.90, −1.98	47%	<0.01	ESWT > LCI
rESWT	4 (238)	−3.11	−6.85, −0.63	60%	<0.01	rESWT > LCI
fESWT	1 (37)	−3.90	−9.26, 1.46	NA	0.15	fESWT = LCI
**VAS**						
Overall	5 (275)	−0.79	−1.12, −0.47	67%	<0.01	ESWT > LCI
rESWT	4 (211)	−0.78	−1.13, −0.43	75%	<0.01	rESWT > LCI
fESWT	1 (37)	−0.90	−1.87, 0.07	NA	0.07	fESWT = LCI
**SNAP**						
Overall	5 (275)	−0.23	−1.14, 0.69	0%	0.63	ESWT = LCI
rESWT	4 (238)	−0.19	−1.11, 0.73	11%	0.68	rESWT = LCI
fESWT	1 (37)	−3.60	−12.77, 5.57	NA	0.44	fESWT = LCI
**CMAP**						
Overall	5 (275)	−0.03	−0.20, 0.13	0%	0.69	ESWT = LCI
rESWT	4 (238)	−0.03	−0.20, 0.13	0%	0.68	rESWT = LCI
fESWT	1 (37)	0.10	−1.99, 2.19	NA	0.93	fESWT = LCI
**SDL**						
Overall	4 (166)	−0.30	−0.35, −0.24	46%	<0.01	ESWT > LCI
rESWT	3 (129)	−0.30	−0.35, −0.25	0%	<0.01	rESWT > LCI
fESWT	1 (37)	0.50	−0.16, 1.16	NA	0.14	fESWT = LCI
**MDL**						
Overall	5 (275)	0.09	0.03, 0.21	0%	0.14	ESWT = LCI
rESWT	4 (238	0.09	−0.03, 0.21	0%	0.16	rESWT = LCI
fESWT	1 (37)	0.30	−0.57, 1.17	NA	0.50	fESWT = LCI
Long-term follow-up (>6 month)						
**BCTQ**						
Overall	2 (74)	−9.45	−14.11, −4.78	0%	<0.01	rESWT > LCI
rESWT	2 (74)	−9.45	−14.11, −4.78	0%	<0.01	
**VAS**						
Overall	2 (74)	−1.65	−2.17, −1.12	0%	<0.01	rESWT > LCI
rESWT	2 (74)	−1.65	−2.17, −1.12	0%	<0.01	

ESWT, extracorporeal shockwave therapy; rESWT, radial extracorporeal shockwave therapy; fESWT, focused extracorporeal shockwave therapy; LCI, local corticosteroid injection; WMD, weighted mean difference; CI, confidence interval; BCTQ, Boston Carpal Tunnel Questionnaire; VAS, visual analog scale; SNCV, sensory nerve conduction velocity; CSA, cross-sectional area of the median nerve.

**Table 3:** Quality of evidence for outcomes and subgroup analyses.

**ESWT for Mild to Moderate CTS**				
**Patient or population:** Patients with mild-to-moderate CTS **Intervention:** ESWT **Comparison:** Other conservative interventions				
**Outcomes**	**Weighted mean difference** **(95% CI)**	**No of Wrists** **(studies)**	**Quality of the evidence** **(GRADE)**	**Comments**
**ESWT versus Control**				
**ΔVAS score 1 month**	The mean ΔVAS score 1 month in the intervention groups was **2.07 lower** (2.30 lower to 1.85 lower)	404 (7 studies)	⊕⊕⊝⊝ **low**	
**ΔVAS score 3 month**	The mean ΔVAS score 3 month in the intervention groups was **1.26 lower** (1.56 lower to 0.97 lower)	379 (6 studies)	⊕⊕⊝⊝ **low**	
**ΔBCTQ score 1 month**	The mean ΔBCTQ score 1 month in the intervention groups was **9.29 lower** (11.57 lower to 7.02 lower)	488 (8 studies)	⊕⊕⊝⊝ **low**	
**ΔBCTQ score 3 month**	The mean ΔBCTQ score 3 month in the intervention groups was **9.47 lower** (7.91 lower to 11.02 lower)	464 (7 studies)	⊕⊕⊝⊝ **low**	
**ΔBCTQ score 6 month**	The mean ΔBCTQ score 6 month in the intervention groups was **15.55 lower** (18.53 lower to 12.57 lower)	119 (2 studies)	⊕⊝⊝⊝ **very low**	
**ΔSNCV 1 month**	The mean ΔSNCV 1 month in the intervention groups was **0.92 higher** (0.55 higher to 1.29 higher)	247 (4 studies)	⊕⊕⊝⊝ **low**	
**ΔSNCV 3 month**	The mean ΔSNCV 3 month in the intervention groups was **1.15 higher** (0.59 higher to 1.72 higher)	267 (5 studies)	⊕⊕⊝⊝ **low**	
**ΔMDL 3 month**	The mean ΔMDL 3 month in the intervention groups was **0.26 lower** (0.35 lower to 0.17 lower)	179 (4 studies)	⊕⊕⊝⊝ **low**	
**ΔCSA 1 month**	The mean ΔCSA 1 month in the intervention groups was **0.55 lower** (0.64 lower to 0.47 lower)	164 (3 studies)	⊕⊝⊝⊝ **very low**	
**ΔCSA 3 month**	The mean ΔCSA 3 month in the intervention groups was **0.85 lower** (0.95 lower to 0.76 lower)	164 (3 studies)	⊕⊝⊝⊝ **very low**	
**ESWT versus LCI**				
**ΔVAS score 1 month**	The mean ΔVAS score 1 month in the intervention groups was **0.02 lower** (0.32 lower to 0.29 higher)	219 (5 studies)	⊕⊕⊝⊝ **low**	
**ΔVAS score 3 month**	The mean ΔVAS score 3 month in the intervention groups was **0.79 lower** (1.12 lower to 0.47 lower)	275 (5 studies)	⊕⊕⊝⊝ **low**	
**ΔVAS score 6 month**	The mean ΔVAS score 6 month in the intervention groups was **1.65 lower** (2.17 lower to 1.12 lower)	74 (2 studies)	⊕⊝⊝⊝ **very low**	
**ΔBCTQ score 1 month**	The mean ΔBCTQ score 1 month in the intervention groups was **0.82 higher** (0.36 lower to 2.01 higher)	219 (5 studies)	⊕⊕⊝⊝ **low**	
**ΔBCTQ score 3 month**	The mean ΔBCTQ score 3 month in the intervention groups was **3.35 lower** (6.28 lower to 0.41 lower)	275 (5 studies)	⊕⊕⊝⊝ **low**	
**ΔBCTQ score 6 month**	The mean ΔBCTQ score 6 month in the intervention groups was **9.45 lower** (14.11 lower to 4.78 lower)	74 (2 studies)	⊕⊝⊝⊝ **very low**	
**ΔSNAP amplitude 1 month**	The mean ΔSNAP amplitude 1 month in the intervention groups was **0.16 lower** (1.19 lower to 0.87 higher)	92 (2 studies)	⊕⊝⊝⊝ **very low**	
**ΔSNAP amplitude 3 month**	The mean ΔSNAP amplitude 3 month in the intervention groups was **0.23 lower** (1.14 lower to 0.69 higher)	275 (5 studies)	⊕⊕⊝⊝ **low**	
**ΔCMAP amplitude 1 month**	The mean ΔCMAP amplitude 1 month in the intervention groups was **0.01 higher** (0.16 lower to 0.18 higher)	145 (3 studies)	⊕⊝⊝⊝ **very low**	
**ΔCMAP amplitude 3 month**	The mean ΔCMAP amplitude 3 month in the intervention groups was **0.03 lower** (0.20 lower to 0.13 higher)	275 (5 studies)	⊕⊕⊝⊝ **low**	
**ΔSDL 3 month**	The mean ΔSDL 3 month in the intervention groups was **0.30 lower** (0.35 lower to 0.24 lower)	166 (4 studies)	⊕⊕⊝⊝ **low**	
**ΔMDL 1 month**	The mean ΔMDL 1 month in the intervention groups was **0.04 higher** (0.11 lower to 0.20 higher)	145 (3 studies)	⊕⊝⊝⊝ **very low**	
**ΔMDL 3 month**	The mean ΔMDL 3 month in the intervention groups was **0.09 higher** (0.03 lower to 0.21 higher)	275 (5 studies)	⊕⊕⊝⊝ **low**	

**ESWT**: extracorporeal shock wave therapy; **CTS**: carpal tunnel syndrome; **CI**: confidence interval; **VAS**: visual analog scale; **BCTQ**: Boston Carpal Tunnel Questionnaire; **SNCV**: sensory nerve conduction velocity; **MDL**: motor distal latency; **CSA**: cross-sectional area; **LCI**: local corticosteroid injection; **SNAP**: sensory nerve action potential; **CMAP**: compound muscle action potential; **SDL**: sensory distal latency. GRADE Working Group grades of evidence; **High quality** (⨁⨁⨁⨁): Further research is very unlikely to change our confidence in the estimate of the effect. **Moderate quality** (⨁⨁⨁⊝): Further research is likely to have an important impact on our confidence in the estimate of the effect and may change the estimate. **Low quality** (⨁⨁⊝⊝): Further research is very likely to have an important impact on our confidence in the estimate of the effect and is likely to change the estimate. **Very low quality** (⨁⊝⊝⊝): We are very uncertain about the estimate.

## Data Availability

Not applicable.
